# Strength and Power-Related Measures in Assessing Core Muscle Performance in Sport and Rehabilitation

**DOI:** 10.3389/fphys.2022.861582

**Published:** 2022-05-02

**Authors:** Erika Zemková

**Affiliations:** ^1^ Department of Biological and Medical Sciences, Faculty of Physical Education and Sport, Comenius University in Bratislava, Bratislava, Slovakia; ^2^ Sports Technology Institute, Faculty of Electrical Engineering and Information Technology, Slovak University of Technology, Bratislava, Slovakia; ^3^ Faculty of Health Sciences, University of Ss. Cyril and Methodius in Trnava, Bratislava, Slovakia

**Keywords:** core muscle tests, core stability, core strength, back pain, force-velocity-power profiling, sport-specific performance

## Abstract

While force-velocity-power characteristics of resistance exercises, such as bench presses and squats, have been well documented, little attention has been paid to load, force, and power-velocity relationships in exercises engaging core muscles. Given that power produced during lifting tasks or trunk rotations plays an important role in most sport-specific and daily life activities, its measurement should represent an important part of the test battery in both athletes and the general population. The aim of this scoping review was 1) to map the literature related to testing methods assessing core muscle strength and stability in sport and rehabilitation, chiefly studies with particular focus on force-velocity-power characteristics of exercises involving the use of core muscles, 2) and to identify gaps in existing studies and suggest further research in this field. The literature search was conducted on Cochrane Library databases, Scopus, Web of Science, PubMed and MEDLINE, which was completed by SpringerLink, Google Scholar and Elsevier. The inclusion criteria were met in 37 articles. Results revealed that among a variety of studies investigating the core stability and core strength in sport and rehabilitation, only few of them analyzed force–velocity–power characteristics of exercises involving the use of core muscles. Most of them evaluated maximal isometric strength of the core and its endurance. However, there are some studies that assessed muscle power during lifting tasks at different loads performed either with free weights or using the Smith machine. Similarly, power and velocity were assessed during trunk rotations performed with different weights when standing or sitting. Nevertheless, there is still scant research investigating the power-velocity and force-velocity relationship during exercises engaging core muscles in able-bodied and para athletes with different demands on stability and strength of the core. Therefore, more research is needed to address this gap in the literature and aim research at assessing strength and power-related measures within cross-sectional and intervention studies. A better understanding of the power-force-velocity profiles during exercises with high demands on the core musculature has implications for designing sport training and rehabilitation programs for enhancement of athletes’ performance and/or decrease their risk of back pain.

## 1 Introduction

The importance of the core for stabilization of the spine and production of a high force in most sports and everyday physical activities has been largely recognized. The “core” is described as a box including the abdominals in the front, the paraspinals and gluteals in the back, the diaphragm at the top, and the hip girdle and pelvic floor muscles at the bottom (Richardson et al., 1999). While core strength is related to strength of the trunk muscles, core stability is defined as the ability to control trunk movements over the pelvis and lower limbs in order to provide conditions for optimal force transfer throughout the kinetic chain to the terminal segment (Kibler et al., 2006).

Exercises for strengthening and stabilization of the core are recommended for rehabilitation and prevention of musculoskeletal disorders, as well as for enhancement of athletic performance (Akuthota and Nadler, 2004; Akuthota et al., 2011; Rivera, 2016; Martín-Moya and Ruiz-Montero, 2017). However, conflicting and limited evidence exists to support their effectiveness. This may be due to non-specific tests used for assessing adaptations of the core after sport training and rehabilitation (Barbado et al., 2016). Evidence is based mainly on the experience of conditioning specialists, the biomechanical analysis of technique or the results of cross-sectional studies. Another shortcoming is that existing testing methods do not focus on the major spine stabilizers despite the fact that the most important stabilizers are task specific (Behm et al., 2010). Low sensitivity and/or validity of laboratory techniques and reliability of field-testing methods assessing the core strength and stability limits their practical applications (Butowicz et al., 2016).

Core strength is usually measured by the number of reps and a load lifted (Faries and Greenwood, 2007). Triaxial back dynamometers are rare (Parnianpour et al., 1988; Gomez et al., 1991; Balague et al., 2010), therefore isokinetic and isometric dynamometers are used (Flory et al., 1993; McGill et al., 1999). The ability to exert trunk muscle force repeatedly or continuously over a long period of time is assessed. A reliable data can be obtained by a 10-min single-session protocol consisting of 4 sets of 15 maximum flexion-extension concentric exertions at angular velocity of 120/s (García-Vaquero et al., 2020). However, the quality of studies evaluating isokinetic strength of the trunk muscles in individuals with acute low back pain (LBP) in comparison with healthy controls is still weak (Reyes-Ferrada et al., 2021).

The National Strength and Conditioning Association (Baechle and Earle, 2002) and the American College of Sports Medicine (Franklin et al., 2000) have recommended the use of core endurance tests. These tests evaluate the endurance of the core muscles (i.e., erector spinae, quadratus lumborum, and external obliques) under static conditions (McGill, 2002; McGill et al., 2003; Faries and Greenwood, 2007). Reduced endurance in these muscles is believed to be associated with LBP (Biering-Sørensen, 1984; McGill et al., 2003; Schellenberg et al., 2007) because they can provide stability and large torques in highly loaded tasks (McGill, 2002; McGill, 2004). The endurance of the anterior, posterior, and lateral muscles of the core as an important component of core stability is assessed using trunk flexion, trunk extension, and right and left side bridge tests (Shamsi et al., 2016). Subjects are required to maintain a neutral-spine position in a supine or quadrupedal posture (Liemohn et al., 2005; Faries and Greenwood, 2007; Gamble, 2007). The side bridge endurance test and the trunk flexor endurance test have high intra-rater and inter-rater reliability, albeit with relatively large standard error of measurement values (Evans et al., 2007). Moreover, the side bridge test can reveal between-group differences in the ratio asymmetry and endurance time (Villalba et al., 2019). A total of 29% of the bridge test performance with unilateral knee extension can be predicted by extensors strength, trunk, and hip internal rotators (Resende et al., 2020).

In addition to static core tests, it is also important to assess the ability of athletes to restore stability following perturbations. Core muscle function plays an important role in controlling intervertebral and global trunk movements. The dynamic core stability also contributes to the control of distal segment movements and loading forces *via* coordinated trunk muscle recruitment (Smith et al., 2008). This muscle coordination and recruitment occurs in response to expected or unexpected perturbations such that proper posture (static stability) or intended movement path (dynamic stability) can be maintained (Smith et al., 2008). Dynamic core stability with integration of upper or lower extremities can be assessed using squat-type movements. Also exercises such as the Olympic clean and jerk weight lift, the lunge or those performed on unstable surfaces require maintainance of the spine and trunk in a stable alignment (Behm et al., 2010).

However, there may be a weak relationship between core stability in dynamic multi-joint and static single-joint exercises because of their high degree of task specificity (Keogh et al., 2010). Therefore, core muscle static tests may be unable to discriminate among power-trained athletes with different performance levels and to reveal the effects of training programs including dynamic core muscle exercises. Tests assessing trunk control during functional activities are limited to linear movements that lack the explosive motions typical for most athletic tasks (Palmer and Uhl, 2011). An appropriate alternative consists of exercises that allow motion in three planes and simulate dynamic movement of the trunk, such as a medicine ball throws (side, overhead, scoop) (Cowley and Swensen, 2008; Kohmura et al., 2008), a diagonal chop and lift exercise using cable pulleys (Palmer and Uhl, 2011), and a modified wood chop exercise using pulley system and an external dynamometer, that is, performed when seated (Andre et al., 2012) or in a standing position (Zemková et al., 2017a). Although the side and front abdominal power tests are easy to perform in the field, most of them are not related to performance measures (Nikolenko et al., 2011).

Besides sport, assessing core muscle strength and endurance is also important in rehabilitation clinical practice. Clinicians use structural and performance testing methods, which may include recording the voluntary surface electromyogram in individuals presenting back pain or recovering from an injury. The structural assessment usually involves a measurement of spinal stability and range of motion (ROM), completed by radiological examination. However, clinicians might fail to diagnose instability of the spine by examination of intervertebral segmental motion and trunk ROM (Binkley et al., 1995; Hicks et al., 2003). In addition, a manual assessment might not reflect movements of the segmental spine *in vivo* (Landel et al., 2008). Furthermore, magnetic resonance imaging might be not sensitive enough in differentiating healthy subjects and those with LBP and spinal abnormalities (Iwai et al., 2004; Okada et al., 2007). Therefore, these structural testing methods can be completed by the performance assessment which allows the tracking of pre- and post-operative rehabilitation progress, as well as predicting the risk of injuries (Flory et al., 1993; Nadler et al., 2000; Nadler et al., 2001; Ireland et al., 2003). Isometric and isokinetic dynamometers assessing the core muscle strength and endurance have been used for this purpose. However, most dynamometers could be expensive, they are not portable for the use in field conditions, and require time-consuming procedures and administration.

In practice, non-dynamometric tests are preferred. The most frequently used is the lumbar extension test by Biering-Sørensen (1984), its variation called the “arch-up test” assessing endurance of trunk extensors, and the flexor and side bridge endurance tests (McGill, 2001). Good isometric muscular endurance has been understood to prevent LBP occurrence (Biering-Sørensen, 1984). However, a recent study by Rausch et al. (2021) reported no correlations between endurance tests that assess an isometric strength of the dorsal, lateral and ventral chains of the core muscles and perceived strength performance, functional status, and self-reported disease-specific pain in individuals with axial spondylarthritis. Furthermore, similar scores in the Bunkie test (a functional performance test consisting of 5 test positions performed bilaterally) were found in subjects with and without a prior history of musculoskeletal injury (Brumitt, 2015). In contrast with non-athletes, male athletes have equivalent holding times on the Biering-Sørensen trunk extensor endurance test to those of female athletes (Evans et al., 2007). However, female athletes have significantly lower holding times on the side bridge endurance tests than their male counterparts (Evans et al., 2007).

Most of these tests evaluate maximum isometric strength of the core muscles and their endurance (e.g., the lateral bridge test, trunk flexor, and extensor endurance tests) rather than muscle power. Given that this parameter is a better predictor of performance in sport and daily life activities, the tests that assesses muscle power of the core may be more appropriate. Therefore, diagnostic systems monitoring basic biomechanical parameters during exercises with high demands on the core musculature should be used to obtain information on power-force-velocity profiles, which may provide basis for designing sport training and rehabilitation programs.

However, a preliminary analysis of the literature indicates that methods assessing strength and power-related measures of core muscle performance are insufficient. The aim of this scoping review was 1) to map the literature related to testing methods assessing core muscle strength and stability in sport and rehabilitation, chiefly studies with particular focus on force-velocity-power characteristics of exercises involving the use of core muscles, 2) and to identify gaps in existing studies and suggest further research in this field.

## 2 Methods

In this paper, designed in the form of a scoping review (Armstrong et al., 2011; Sucharew and Macaluso, 2019), two questions were addressed: 1) Which methods are used and related variables analysed during exercises engaging core muscles? 2) Do current tests provide sufficient information on force-velocity-power characteristics of the core musculature?

An electronic literature search was provided to analyse existing research dealing with core stability and core strength testing within cross-sectional and intervention studies. The literature search was conducted on Cochrane Library databases, Scopus, Web of Science, PubMed and MEDLINE, and completed by SpringerLink, Google Scholar and Elsevier. Articles in peer-reviewed journals were considered for analysis. References in reviews were searched manually to identify further relevant articles. If overlapping data resulting from similar or the same studies were included in multiple papers, the one with the most recent publication date was analyzed. Articles or abstracts published in conference proceedings, theses, case studies, and books were excluded. Articles were also excluded if they did not contain original research or were incomplete. The inclusion criteria involved research articles that specified participants, experimental protocols, and measures relevant to the scope of this review. Literature searches were limited to English language. Papers published after 1990 were preferred, however earlier relevant studies were also included. Articles that failed to meet the eligibility criteria for this scoping review were excluded.

The initial search was confined to research papers that were close to the main purpose of this scoping review, i.e., those investigating force-velocity-power characteristics of exercises involving the use of core muscles (e.g., lifting tasks, and trunk rotations). However, this approach revealed only a limited number of papers that met the eligibility criteria. The search was therefore widened to include investigations dealing with testing methods assessing not only muscle power but also maximal isometric strength and endurance of the core muscles. These together helped to identify gaps in existing studies and formulate recommendations for further studies on this topic.

**FIGURE 1:**
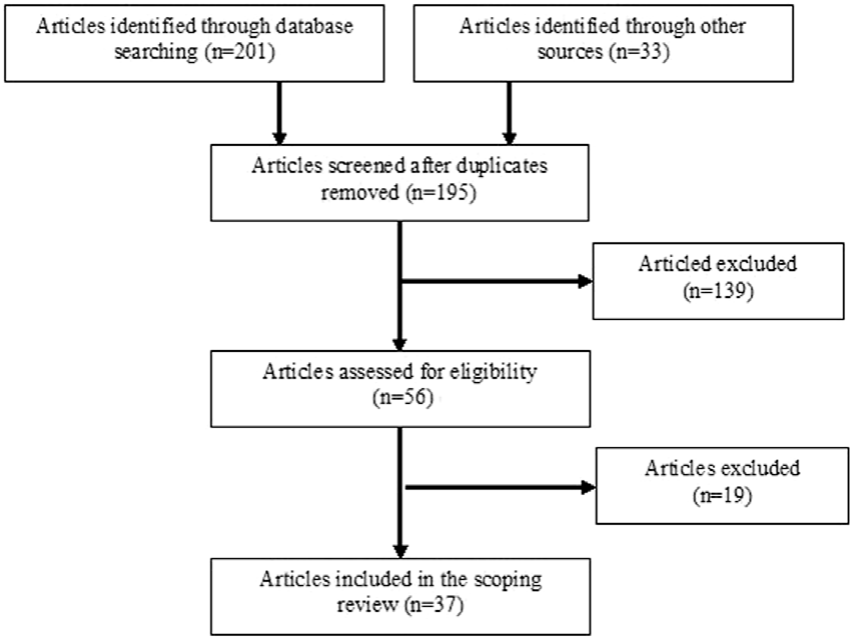
A flowchart showing phases of the literature search.

The search and appraisal of selected studies on the basis of exclusion and inclusion criteria was performed by the author of this review. Some concerns were related to sample representativeness, missing information about the control group and/or non-controlled compliance of experiments, as well as cases where testing methods and variables were not precisely described. The target population was athletes of team and individual sports with high demands on the core musculature. Proposed sports (i.e., baseball, hockey, table tennis, tennis, golf, boxing, thai boxing, karate, tae kwon do, judo, wrestling, bodybuilding, weightlifting, ballroom dancing, rock and roll dancing, canoeing, kayaking, rowing) were combined with the following keywords. A combination of these terms was included in the search strategy: “core muscles” AND “core muscle test” AND “core stability” AND “core strength” AND “core training” AND “force” AND “lifting task” AND “maximal isometric strength” AND “muscular endurance” AND “power” AND “trunk rotations” AND “velocity.” Further searches were conducted using words from subheadings that, for example, specified resistance exercises engaging core muscles. Altogether 201 articles were identified through database searching. Following an initial screening and assessing for eligibility, papers were removed if they failed to meet the inclusion criteria. 37 articles that addressed methods assessing core stability and core strength in sport and rehabilitation were analysed. Figure 1 includes phases of the search procedure.

## 3 Results and Discussion

### 3.1 Assessing Muscle Strength and Power During Exercises Engaging Core Muscles

The analysis of the literature revealed that most studies have evaluated maximal isometric strength and endurance of the core muscles rather than muscle power (Supplementary Table S1). Actually, only a few studies have analysed power-velocity-force characteristics of exercises involving the use of core muscles.

#### 3.1.1 Assessing Muscle Strength and Power During Lifting Tasks

So far, maximal isometric strength measurements of the back muscles have been recommended for assessing performance in lifting tasks (Karwowski and Marras, 1999). An inadequate isometric strength was assumed to be associated with LBP. However, there is a threefold increase in the risk of chronic back problems when the demands of a lifting task is equal to or beyond an individual’s muscle strength capacity. The load on the spine during a lifting task is considerably underestimated during muscle strength measurements under static conditions. A load on the spine is predicted to be 33%–60% (depending on the lifting technique) higher under dynamic than static conditions. The internal load on the spine and the recruitment pattern of trunk muscles differ substantially in these two conditions. However, it may also depends on joint angles used during isometric maximum strength assessment. For instance, maximum torques of the lower body differ significantly between contraction types, and this may be specific to the joint action, muscle groups used and the context of the testing (Stotz et al., 2022). Maximum isometric torque can exceed eccentric and concentric torque in lower extremity joint actions when joint angles are individually adjusted (Stotz et al., 2022). The knee and hip angles of 141° and 124° respectively used for MVC assessment of the back muscles correspond to the portion of the clean lift where the highest power is produced (Garhammer, 1993). Nonetheless, moderate to low relationships between lifting task capacity and maximum isometric muscle strength indicate that lifting performance cannot be based only on muscle strength testing under static conditions (Rosecrance et al., 1991). Performing dynamic lifting tasks seems to be a more appropriate method for LBP individuals.

An exercise engaging major muscle groups, such as the lower and upper back, erector spinae, abdomen, gluteus maximus, quadriceps, and hamstrings (i.e., deadlift to high pull) may at best mimic the requirements of jobs and sports involving a lifting task. The exercise is usually performed with stepwise increasing loads either with free weights or using the Smith machine. The power increases from lower loads, reaches a maximum (mean at ∼70% 1RM and peak at ∼80% 1RM), and then toward higher loads, decreases again. However, the power is significantly higher when the exercise is performed with free weights compared to using the Smith machine at loads ≥50 kg (Zemková et al., 2016). Taking into account sufficient reliability (ICC above 0.80 for both peak and mean power) and stability of measurement (no significant between-session differences in their values) (Zemková et al., 2016), the deadlift to high pull exercise can be applied for assessing muscle power during lifting tasks. Mean rather than peak power should be used for data analysis because it represents a more reliable parameter (SEM <10% and >10%, respectively) (Zemková et al., 2016). The movement pattern during the exercise with free weights seems to be closer to the lifting demands of many sport and daily life activities than the one performed on the Smith machine, plus it can be more easily implemented in the practice because a weight stack machine is not required.

Furthermore, this test is sensitive in differentiating the power performance between physically active and sedentary young adults at higher weights (≥45 kg). Significant individual differences in power and velocity during the deadlift to high pull have been observed at a load of maximal power achieved. Its peak and mean values are achieved at higher weights in physically active (about 79% and 86% 1RM) than sedentary young adults (about 71% and 79% 1RM). These between-group differences in power are observed during the upright row but not during the deadlift. The power during the deadlift to high pull correlates significantly with the one produced during the upright row. It also depends on how much experience individuals have with the deadlift technique. Trunk angular acceleration and vertical bar acceleration is maximal near lift-off in the skilled lifters, whereas the unskilled individuals demonstrate higher magnitude and variability in angular and linear acceleration variables (Brown and Abani, 1985). From a practical point of view, analysing maximal values of power during lifting tasks seems to be a more appropriate alternative than the use of 1RM. Three months of resistance training enhanced power outputs during a lifting task in the form of a deadlift high pull with free weights from 30 to 50 kg (∼40%–60% 1RM) in overweight and obese individuals (Zemková et al., 2017c).

However, one has to be careful when this exercise is performed by sedenatry individuals, in whom the use of higher additional loads should be avoided. The loaded lifting task should be performed with caution also in whose prone to LBP. An alternative is represented by the test assessing the ability of individuals to develop a high force in a short period of time during MVC of the back muscles. This assumption is based on a significant correlation between power during the deadlift to high pull at light loads and peak rate of force development (RFD) during MVC of the back muscles (Zemková et al., 2019b). This indicates that peak RFD measured during MVC of the back muscles might predict a lifting performance at light loads. MVC peak force also significantly correlates with the power produced during a loaded lifting task, however at higher weights. It means that a greater isometric back muscle strength is needed for power generation at heavier loads.

Another promising method for LBP individuals requires to estimate pre-determined percentage (e.g., 50%) of MVC of the back muscles. It assesses their ability to differentiate the strength of back muscle contraction either with or without feedback information on force produced under fatigue and non-fatigue conditions. Force feedback contributes to a more accurate force regulation during MVC of the back muscles under fatigue (Zemková and Jeleň, 2020).

#### 3.1.2 Assesssing Muscle Strength and Power During Trunk Rotations

Core muscle strength plays a significant role in transfering torques and momentum throughout the kinetic chain to the extremities (Shinkle et al., 2012). Impairments of this kinetic chain may affect performance or increase the risk of injury (Behm et al., 2010). It is therefore necessary to take into consideration the demands of joints and muscles proximal and distal to the core in this kinetic chain when assessing core muscle strength. Core muscle endurance and strength of upper and lower limbs are related to each other, thus they must be trained and tested as a whole (Kocahan and Akınoğlu, 2018).

The importance of the core musculature was demonstrated for various tasks, for instance pitched ball velocity in baseball pitchers (Stodden et al., 2001; Aguinaldo et al., 2007), and forehand and backhand strokes in tennis players (Ellenbecker and Roetert, 2004). Throwing velocity correlates also with lateral to medial jumps in baseball players (Lehman et al., 2013). Further, a high correlation was reported between handball-throwing speed and throwing with a light but not with a heavy medicine ball (Rivilla-Garcia et al., 2011). According to the authors general tests have limited applications in testing the specific throwing performance (Rivilla-Garcia et al., 2011). There is a small shared variance of the scoop medicine ball throw with baseball fielding (agility T-test, standing long jump, and throwing distance) when compared to batting (Kohmura et al., 2008). The association of throwing velocity with muscle power highlights its importance for performance in cricket players (Freeston et al., 2016). However, Talukdar et al. (2015) suggests that power obtained from the chop and lift exercise is not an important contributor to throwing velocity in cricket.

Effective execution of the golf swing or tennis stroke requires both high limb movement speed and great power production during trunk rotations. Trunk rotators, flexors, extensors, and lateral bend agonists are all involved in the stroke in tennis and baseball or the golf swing. In particular, these muscles are active in the acceleration phase of the golf swing with the highest level of activity observed in the trail-side abdominal oblique muscles (Watkins et al., 1996). However, abdominal strength measured by an isometric abdominal test does not depend on playing position and/or the sports level in professional soccer players (Michaelides et al., 2019).

In the laboratory, isokinetic machines (Newton et al., 1993; Kumar et al., 1995; Kumar, 1997) and electromyography (Pope et al., 1986; McGill, 1991; Kumar and Narayan, 1998) have been used to assess muscle strength during axial rotations. Isokinetic dynamometers, however may be not sensitive enough in revealing between and within-group differences. For instance, the trunk muscle endurance and peak torque in elite golfers does not differ significantly from non-golfers (Lindsay and Horton, 2006). Similarly, a decline in the electromyography median frequency and the static holding times in low-handicap golfers with LBP does not differ significantly from healthy non-golfers (Suter and Lindsay, 2001). The limitation is that rotations performed with a torso and legs attachment while sitting on a chair represent a non-specific movement pattern. The external validity of isokinetic and isometric trunk muscle strength tests for functional activities is ambiguous. Tests that closely mimic movements specific to a particular sport or daily life activities are more appropriate. For instance, the diagonal chop and lift mimic tasks which require dynamic control of the trunk. While the side-plank and the chop and the lift tests moderately correlate, there is a low correlation between the Biering-Sørensen test and power measurements (Palmer and Uhl, 2011).

Novel torso isoinertial dynamometers allow assessing velocity and power during trunk rotations. Test-retest reliability of these parameters is good to excellent (ICC >0.90, SEM ∼7%) when a weight of 1 kg is used; however their values should be interpreted with caution when the exercise with a barbell of 20 kg is performed (ICC <0.80, SEM >10%) (Zemková, 2019). This measurement is also sensitive in discriminating trunk rotational velocity in athletes of different sports. Its values are the highest in canoeists, then ice-hockey players, rock and roll dancers, judoists, wrestlers, tennis players, golfers, karateists, and finally ballroom dancers. More specifically, tennis players perform better than golfers, rock and roll than ballroom dancers, whereas judoists and wrestlers do not differ significantly (Zemková et al., 2013). Similarly, greater power is produced in tennis players than golfers, rock and roll than ballroom dancers, and judoists than wrestlers. Individual comparisons of these parameters revealed higher values in a weightlifter than a bodybuilder, a canoeist than a rower, and an ice-hockey player than a karateist. These between and within-group differences in velocity and power during rotational movements of the trunk may be ascribed to training specificity including trunk rotations at different velocities under different load conditions.

However, it can be more appropriate to use the weight stack machines when testing athlete performance as compared to frequently used dynamometers allowing trunk movements in a fixed seated position. A device which determines kinetic characteristics during rotational movement of the trunk provides conditions specific to the demands of many sports. The simultaneous use of an external dynamometer and a pulley system represents a reliable tool for testing muscle power during a rotational exercise of the axial skeleton in the transverse plane while sitting on a box (Andre et al., 2012).

This measurement may be suitable for canoeists or kayakers, however for other athletes, such as tennis or hockey players, trunk rotations in a standing position may be a more appropriate alternative. The test adapted from the woodchop exercise most likely provides conditions close to those imposed in sporting activities including rotational movements of the trunk (e.g., baseball, golf, hockey, karate, tennis, etc.). It allows assesssing both muscle power at different loads and muscular endurance during repeated standing cable woodchop exercises. Mean power is a reliable (ICC above 0.90) and sensitive parameter in revealing between and within-group differences (Zemková et al., 2017a). Significant individual differences have been observed mainly at higher weights (>65% 1RM), at which maximal values of power are usually achieved. Angular velocity has influence on the axial rotation torque and electromyographic activity of trunk muscles (Fan et al., 2014). There is an inverse relationship between these measures (Fan et al., 2014). Higher angular velocity is related to greater coactivation of antagonist muscles that leads to a torque reduction with increasing velocity (Fan et al., 2014). The largest muscle activities during rapid, three-dimensional pulling tasks occur in muscles with the highest spatial efficiency producing the task moments (Thelen et al., 1996). However, latissimus dorsi and abdominal oblique muscles are active during pulling tasks that involve lateral or axial moments in spite of poor spatial efficiency to equilibrate the task moments (Thelen et al., 1996).

Although computer-based diagnostic systems attached to weight stack machines can be used for fitness-oriented testing of muscle power during trunk rotations, practitioners prefer to use free weights for their workouts. The reason is that machines can neglect crucial stabilization components of the core musculature. Exercises with free weights place higher demands on core stabilizers and allow performing a full trunk ROM. They also better mimic most sport and daily living tasks, are less expensive than weight machine exercises and can be used in the sporting field.

Therefore exercises that simulate rotational movements of upper and lower body should be used in functional assessment of sport-specific performance. One can measure peak and/or mean torque, force, velocity, and power in both acceleration and deceleration phases of trunk rotations either while sitting or standing with a barbell placed on the shoulders. There are significant correlations between values measured in these phases at loads ranged from 1 to 20 kg (mean power from −0.77 to −0.92, mean force and mean torque from −0.56 to −0.78, mean velocity from 0.64 to 0.84). This indicates that muscle power does not differ significantly between these two phases, regardless of a load used, when powerful trunk rotational movements are performed with aim to develop high velocity over the entire ROM.

Performing single repetitions of trunk rotations with increasing loads stepwise up to the 1RM allows designing power-velocity and force-velocity curves and/or analysing velocity and power to weight used relationship. It is well known that peak force occurs when the movement speed is very low. As the movement speed increases, force decreases and is very low at very high speeds. Maximal power during trunk rotations is achieved at weights of 30%–45% 1RM, which is lower compared to values achieved at intermediate velocities when lifting weights of 50%–60% 1RM during resistance exercises (e.g., bench presses and squats). Variations in power production in athletes of different sports may be attributed to adaptations specific to the training undertaken. This assumption may be corroborated by significant differences between groups of athletes in power generated during trunk rotations at lower velocities (≤334.2 rad/s) or at higher weights (≥10.5 kg). The highest power is achieved in combat sports athletes (maximum at 10.5 kg), then in water sports athletes (maximum at 20.0 kg), grappling sports athletes (maximum at a 15.5 kg), and ball sports athletes (maximum at a 10.5 kg) (Zemková et al., 2020). Additionally, angular velocity is the highest at lower weights in combat sports athletes and at higher weights in water sports athletes. Alternatively, combat sports athletes produce the highest force at higher velocities, whereas water sports athletes produce the highest force at lower velocities. While the highest power is produced at higher velocities or at lower weights in ball sports players (golf, hockey, tennis) and combat sports athletes (tae kwon do, thai boxing, karate, boxing) who generate high force in a short period of time, the highest power at higher weights is produced in grappling sports athletes (judo, wrestling) who require a great explosive power of upper and lower body to lift and throw the opponent and water sports athletes (canoeing, kayaking) who exert a great force against the water. These variables are able to discriminate groups of athletes with different demands on production of velocity and power in unloading and loading conditions, and may also reflect their training specificity.

The ability to generate maximal power is influenced by the type of muscle action involved and, in particular, the time available to develop force, storage and utilization of elastic energy, interactions of contractile and elastic elements, potentiation of contractile and elastic filaments as well as stretch reflexes (Cormie et al., 2011). Furthermore, maximal power production is influenced by morphological factors including fibre type contribution to whole muscle area, muscle architectural features and tendon properties as well as neural factors including motor unit recruitment, firing frequency, synchronization and inter-muscular coordination (Cormie et al., 2011). Thus, the magnitude of the athlete-related differences in strength and power outputs may depend on differences in muscle cross-sectional area, fibre type distribution, the muscle mechanics and their training background (Izquierdo et al., 2002). It is most likely that training with loads that maximize power output elicits specific neural and muscle fibre adaptations (i.e., increasing the number of type II fibres) (Cormie et al., 2011). An understanding of physiological mechanisms of maximal power production is therefore importatnt for designing effective training programs for its enhancement. Strength training prescription depends on the sport-specific requirements and the type of standing and seated trunk rotations performed with or without external resistance. Developing of trunk rotational power in ball, combat, grappling, and water sports athletes requires the individualised “optimal” load and the number of repetitions, among other factors. Maximal values of power usually occur at light to moderate weights (i.e., at 15 and 10 kg in most athletes, in few of them at 20 kg or even at 25 kg) when trunk rotations with maximum efforts are performed. Individually adjusted load conditions allow to perform trunk rotational movements with similar velocities to those encountered in a particular sport. Repetitions performed above 90% of peak power with a particular weight are the most efficient for maximizing power improvements. This provides better conditions for contributing of each neuromuscular factor to the individual athlete’s adaptation.

The asymmetric trunk loading in some sports (e.g., tennis, golf) may lead to between-side imbalances in strength and endurance of the core muscles. The compression on the spine is higher during asymmetric than symmetric pulls that impose twisting loads on it, and require additional efforts to equilibrate the twisting torques (Thelen et al., 1996). These imbalance can increase the risk of LBP and back injuries. They may account for 5%–25% of all injuries in tennis and 15%–34% in golf. However, only a small number of LBP indicators has been identified. For instance, muscular endurance in the non-dominant side (the follow-through of the golf swing) is significantly lower in golfers with LBP than in healthy subjects (Lindsay and Horton, 2006). Dysfunction exists when the left and right side scores in the time which the subject can hold the sidelying position differ by more than 5%. On the contrary, there are no significant differences in peak torque between dominant and non-dominant rotation within any group (control normals, control golfers, and golfers with LBP) (Lindsay and Horton, 2006).

However, when peak and mean power were evaluated during trunk rotations with an additional load (a barbell placed on the shoulders) while standing, their values were significantly lower on the non-dominant than dominant side in tennis players (∼14.6% at 5.5–15.5 kg), ice-hockey players (∼15.25% at 5.5–20 kg), and golfers (∼17.75% at 5.5–10.5 kg), there were no significant differences in the age-matched control group of physically active individuals (<10%) (Zemková et al., 2019a). Similarly, backhand rotation strength was found to be slightly greater than forehand rotation (by 4%–8%) in elite female tennis players, whereas trunk rotation strength is symmetric in elite male tennis players (Ellenbecker and Roetert, 2004). Conditioning programs for asymmetric sports should be designed to reduce between-side differences in trunk muscle strength and power. Investigating the trunk rotational strength asymmetry may be particularly important for young individuals, for instance adolescents with idiopathic scoliosis (McIntire et al., 2007).

Besides young competitive athletes, also older adults take part in sporting activities involving loading and unloading trunk rotations (e.g., canoeing, golf, table tennis, tennis, etc.). It is likely that increased trunk stiffness with age (Gill et al., 2001; McGibbon and Krebs, 2001; Allum et al., 2002) may contribute to lower trunk ROM, and consequently also angular velocity and muscle power generated during trunk rotations. Indeed, there are significantly lower values of velocity and respective angular displacement during trunk rotations in older than young adults (Zemková et al., 2018a). In addition, a relationship exists between angular velocity and trunk ROM in both older (0.772–0.927) and young adults (0.650–0.790). This indicates that lower angular velocity is very probably due to limited trunk ROM, particularly in older subjects. However, Talukdar et al. (2015) reported that higher ROM at proximal segments (i.e., thoracic and hips) does not lead to higher throwing velocity in cricket because lower ROM at proximal segments can contribute to the momentum transfer from the lower limbs in such explosive tasks.

Nonetheless, a limited trunk ROM, in addition to decreased posterior concavity, most likely contributes to slower velocity of trunk rotations in wheelchair athletes. Lumbar inversion and pelvic retroversion are lower in para table tennis players than in able-bodied athletes, whereas thoracic kyphosis values are similar in both groups. In addition, para table tennis players generate significantly lower velocity concomitant with smaller respective angular displacement during trunk rotations than able-bodied athletes. Velocity in the acceleration phase of trunk rotation is associated with angular displacement in both groups, whereas with lumbar curvature and pelvic tilt angle only in wheelchair athletes (Zemková et al., 2018b).

Trunk rotations performed in a sitting position reduce the involvement of lower limbs and thoracic/hip mobility to the power generated by the upper body. Reduced thoracic spine and hips ROM, that allow the highest rotations because of the joints orientation (Sahrmann, 2002), may affect velocity of trunk rotations and subsequent velocity of the ball in striking and throwing sports. These activities require explosive movement production in the oblique or transverse planes (Earp and Kraemer, 2010). The force is transferred from proximal (i.e., hips) toward distal segments (i.e., shoulders and arms). Because of the kinetic chain between these segments (Putnam, 1993), it is most likely that rotational mobility plays a crucial role in power production during trunk rotations. This power transfer from lower limbs to upper body is important for production of greater throwing velocity.

The core musculature facilitates trunk movements more easily when rotations are performed in an upright position. The power produced is greater compared to those performed in a sitting position, particularly at higher weights (≥10.5 kg) (Zemková et al., 2017b). A greater trunk ROM during standing than seated trunk rotations allows individuals to accelerate the movement more forcefully in an initial phase of rotations. This results in higher velocity and overall power outputs. However, standing trunk rotations that involve the lower body are less confined to the trunk. A weak relationship between power measured during standing and seated trunk rotations with additional loads of ≥10.5 kg indicates that each of them assess distinct qualities. This fact should be taken into consideration when muscle power under these conditions is assessed. Exercises that closely replicates the upper/lower body rotational movements in a particular sport should be preferred in testing in order to assess power under sport-specific conditions. Mean power in the acceleration phase of trunk rotations is significantly higher in standing compared to a sitting position at weights of 10.5–25 kg (from 20.4% to 27.1%) but not at 5.5 kg (13.6%) in athletes that use to perform standing trunk rotational movements in their sports, such as boxers, hockey players, judoists, karateists, tennis players, and wrestlers. However, its values do not differ significantly during standing and seated trunk rotations in canoeists and kayakers at weights of 10.5–25 kg (from −4.6% to 5.1%). In other words, trunk rotational power is significantly higher in athletes performing seated than standing trunk rotations in their sports when measured in a sitting position with weights from 10.5 to 25 kg but not with 5.5 kg. However, its values do not differ significantly between these two groups of athletes when measured in a standing position at weights of 5.5–25 kg. These between-group differences in power during standing and seated trunk rotations are most likely due to a predominant exercise mode used during athletic performance.

Furthermore, the importance of power to weight as a determinant of performance in sports involving trunk rotations has to be taken into account, especially when exercising in a standing position with higher loads. There are poor and moderate correlations between the body weight and work or torque produced at both the dominant and non-dominant side in golfers during isokinetic torso rotation measurements in a sitting position (Lindsay and Horton, 2006). Non-significant correlations are also between the body weight and muscle power during seated trunk rotations using free weights. Similar relationships exist between these variables during standing trunk rotations with lower weights. However, significant correlations between the body weight and trunk rotational power with weights ≥15 kg indicate the importance of determining its relative values when it is tested in a standing position. The power expressed relative to body weight seems to be more important measure than absolute power for most athletes to consider, namely for those whose performance depends heavily on rotational movements of the trunk while standing.

#### 3.1.3 Core Muscle Strength and Power Assessment in Sport and Rehabilitation

Overall, the research in this field is mainly focused on associations of strength and/or endurance of the core muscles with different abilities, such as hand-eye coordination in non-athletes with LBP (Reddy et al., 2017), knee valgus during single-leg squat in male junior athletes (Affandi et al., 2019), agility in professional basketball players (Cengizhan et al., 2019), scapular muscle endurance in professional athletes of basketball, football and handball (Cobanoglu et al., 2019), and balance in subjects with osteoarthritis knee (Joshi et al., 2019). The McGill test and double leg lowering test correlate with performance in the vertical jump, 40 yard dash, medicine ball throw and T tests significantly more than with 60-s sit-ups, indicating that core muscle endurance is important for optimal athletic performance (Shaikh et al., 2009).

While some authors have shown the relationship of core muscle strength with variables of athletic performance (Brown and Abani, 1985; Abt et al., 2007; Sato and Mokha, 2009; Sharrock et al., 2011), others have not (Stanton et al., 2004; Tse et al., 2005). When such correlations were found, most of them were weak or negligible (de Bruin et al., 2021). For instance, core stability was found to be moderately correlated with performance and strength in division I football players (Nesser et al., 2008). Further, a modified plank test correlates with flexor isokinetic trunk muscle strength at the velocity of 60°/sec, and Oswestry Disability Index score negatively correlates with flexor isokinetic trunk muscle strength at the velocity of 180°/sec in amputee soccer players (Aytar et al., 2012). A systematic review by Prieske et al. (2016) indicates that strength of the trunk muscles plays a minor role in athletic performance and physical fitness in trained subjects. Though core strength training increases strength of the trunk muscles, it is associated with limited gains in measures of athletic performance and physical fitness in comparison with regular training (Prieske et al., 2016). Also a critical review by Silfies et al. (2015) indicates that there is a limited evidence supporting the application of core stability training for enhancement of athletic performance and injury prevention. Specific performance tests are needed to better reveal the relationship of core stability with athletic performance (Sharrock et al., 2011).

Several authors have investigated the effects of core muscle training programs on a variety of characteristics of physical fitness. Most studies were related to muscle strength and power, such as leg muscle strength, abdominal muscle strength, and balance in male students of physical education and recreation health (Firdauz and Setijono, 2018), core muscle strength and endurance in school’s football team players (Boyaci and Tutar, 2018), isometric leg and back muscle strength, abdominal muscle strength, back extensor endurance, dynamic balance, flexibility of the lower back and hamstring muscles, thoracic and lumbar spine ROM and lateral bending in male university students (Yaprak, 2018), balance, agility and explosive force in runners (Dinç and Ergin, 2019), neuromuscular control of the trunk and lower limbs during jump landing and single-legged squatting in female collegiate basketball players (Sasaki et al., 2019), core muscle strength in young male cyclists (Chok, 2020), core muscle strength in professional football players (Etxaleku et al., 2020), core muscle strength in junior swimmers (Marani et al., 2020), endurance, strength and balance in female students with trunk defects (Mohebi Rad and Norasteh, 2020), core muscle performance in rhythmic gymnasts (Esteban-García et al., 2021), and neuromuscular control and strength of the trunk muscles in pediatric soccer players (Kumahara et al., 2021). Other studies were related to balance, endurance and flexibility, for instance endurance, strength, flexibility and balance in sedentary women (Sekendiz et al., 2010), dynamic balance, spinal mobility, functional mobility and trunk muscle strength in older adults (Granacher. et al., 2013), trunk muscular endurance in school-aged children (Allen et al., 2014), and balance in adolescent taekwondo athletes (Tayshete et al., 2020). Some of them were also related to speed and agility, for example, throwing velocity in female handball players (Saeterbakken et al., 2011), spinning wheel kick, balance, core strength, power and reaction speed in young female karateists (Kamal, 2015), and smash stroke performance and dynamic balance in badminton players (Hassan, 2017). Remaining studies were related to other abilities, including lower-extremity stability, 5000-M performance and running kinetics in runners (Sato and Mokha, 2009), running performance and economy in runners (Tong et al., 2016), and core muscle endurance and running economy in male college long-distance runners, football, basketball, and rugby players (Hung et al., 2019). Other authors have studied the effectiveness of various training programs on core strength, core stability and/or core/torso stiffness, such as isometric and dynamic core training (Lee and McGill, 2015), foam rolling and core stabilization training (Junker and Stöggl, 2019), dynamic Swiss ball training (Nuhmani, 2021), and so forth.

However, only few studies have analysed exercise-induced changes in power-related measures. For instance, a 9-week isokinetic training program in pre-elite golfers improved ball speed, peak arm speed and acceleration, carry distance, rotational power and force more than isotonic training (Parker et al., 2017). Similarly, an 8-week core strength training program improved rotational power, time to peak acceleration, maximal countermovement jump, estimated peak power, core strength, and rotational flexibility in junior competitive surf athletes (Axel et al., 2018). Another study revealed a significant increase of power in the acceleration phase of trunk rotations after both preparatory (at weights from 10 to 26 kg) and competitive training periods (at weights from 6 to 26 kg) in tennis players (Poór and Zemková, 2018). Mean power significantly increased also in ice-hockey players after the preparatory (at weights ≥12 kg) but not after the competitive period (Poór and Zemková, 2018). Likewise its values significantly increased in canoeists after the preparatory period only (at weights ≥10 kg) (Poór and Zemková, 2018). Similar pre-post training changes were found in the case of peak power (Poór and Zemková, 2018). This indicates that muscle power obtained during trunk rotations is able to reflect their training specificity. It also provides useful information for designing training programs aimed at improvements of trunk rotational power under load conditions. Specific trunk stability adaptations are induced by sport-specific training, which cannot be revealed by using non-specific tests (Barbado et al., 2016). The authors found that kayakers and judokas perform better than recreational athletes in tests that reflect their specific demands (Barbado et al., 2016).

A better understanding of the role of core stability and/or core strength in sport and daily life activities has not only implications for their enhancement but also for decreasing the risk of back pain. In particularly, repeated or prolonged back flexion may be associated with back pain and related risk of injuries (McGill, 2010). A large number of continuous bending cycles may have a detrimental effect on spinal tissues (Contreras and Schoenfeld, 2011). Intradiscal pressure is highest in flexion and lowest in lateral bending (Schmidt et al., 2007). A combination of lateral bending plus flexion or lateral bending plus extension strongly increases the maximum shear strains (Schmidt et al., 2007). Lateral bending plus axial rotation yields the highest increase in fiber strains, followed by axial rotation plus flexion or axial rotation plus extension (Schmidt et al., 2007). The highest shear and fiber strains are both located posterolaterally (Schmidt et al., 2007). An additional axial preload tends to increase the pressure, the shear, and fiber strains (Schmidt et al., 2007). The different physiological loadings in the sports like cricket, field hockey or basketball play an important role in the development of degenerative changes of the lumbar spine, which may be considered a risk factor for future injury and/or LBP in each specific sport because of the unique demands of each discipline (Rozan et al., 2016).

One of the areas of the body which is very often injured by athletes is the lower back (Alexander, 1985; Dunn et al., 2006; Rozan et al., 2016). The type of lumbar spine injuries depends on the direction, magnitude, and the point of application of the forces to the spine (Alexander, 1985). The most common types of lower back injuries in athletes include muscle strains, ligament sprains, lumbar vertebral fractures, disc injuries, and neural arch fractures (Alexander, 1985). The most common serious injuries are neural arch fractures at the pars interarticularis, or the isthmus between the superior and inferior articular processes (Alexander, 1985). These fractures are known as spondylolysis, or defect in the pars interarticularis of one side of the vertebrae; and spondylolisthesis, a bilateral defect in the pars interarticularis, often accompanied by forward displacement of the vertebral body (Alexander, 1985). The lumbar spine with a unilateral pars defect is able to maintain spinal stability as the intact lumbar spine, but the contralateral pars experiences greater stress (Wang et al., 2006). For the lumbar spine with a bilateral pars defect, the rotation angle, the vertebral body displacement, the disc stress, and the endplate stress, increase more when compared to the intact lumbar spine under extension or torsion (Wang et al., 2006). A recent literature review by Tawfik et al. (2020) revealed that the incidence of pars interarticularis defects is the highest in diving, cricket, baseball/softball, rugby, weightlifting, sailing, table tennis, and wrestling. The pars interarticularis of each vertebra is vulnerable to injury if repetitive flexion, rotation and hyperextension are present in the activity, for instance during fast bowling (Elliott, 2000). Hyperextension and repetitive microtrauma in young gymnasts also lead to vertebral injuries, which range from stress reaction to spondylolisthesis (Ciullo and Jackson, 1985). The strength prediction may be employed to quantify the risk of fracture in physically very demanding tasks (Brinckmann et al., 1989). A strength prediction of all vertebrae of an individual spine can be based on the density and area measurement of only one vertebra, and strength of the adjacent vertebrae may then be extrapolated with high accuracy (Brinckmann et al., 1989).

Among the sports in which lower back injuries commonly occur, gymnastics, weightlifting, and football are at greatest risk (Alexander, 1985). To reduce the high incidence of injuries to this part of the body, athletes should increase the strength of the abdominal muscles, and to maximize the flexibility of the lower back (Alexander, 1985). A lesser frequency of LBP is associated with good core strength of the dorsal muscle chain (Ruckstuhl and Clénin, 2019). The exercise programs should be designed to ensure progressions beginning with corrective and therapeutic exercises through stability/mobility, endurance, strength and power stages (McGill, 2010). Motor control exercises combined with non-machine-based resistance exercise, as well as machine-based resistance exercises, increase lower trunk muscle size (Shahtahmassebi et al., 2014). However, there is no effect of non-machine-based resistance and cardiovascular exercises on trunk muscle morphology (Shahtahmassebi et al., 2014). Core strengthening and stabilization exercises may be also beneficial for disc health (Contreras and Schoenfeld, 2011). There is no evidence that a low volume, strength-based exercise routine that includes dynamic spinal flexion movements hasten the onset of disc degeneration (Contreras and Schoenfeld, 2011). Maintaining proper posture and providing correct exercise techniques may help to prevent exacerbation of back problems. For instance, the lumbar lordotic posture is recommended when lifting from the floor level (Hart et al., 1987). It appears that the combination of anatomical design and neural control of the musculature leads to a situation where the resultant shear force on the joint can be maintained at a relatively constant and safe level during dynamic squat lifts (Potvin et al., 1991). This “safety” mechanism is useful only with the preservation of lordosis during lifting, when the muscles must provide the majority of the support moment (Potvin et al., 1991). Furthermore, using a specific pattern in throwing can contribute to the improvement of pitching performance in athletes and decreasing their risk of overuse injury (Aguinaldo et al., 2007). The rotation of baseball pitchers can be optimized by conserving the momentum produced by the trunk when moving the throwing shoulder with decreased joint loading (Aguinaldo et al., 2007). Moreover, cycling mechanics may be altered by core fatigue and consequently increase the risk of knee injuries (Abt et al., 2007). As the core is relatively resistant to fatigue, improved core muscle endurance and stability may promote greater alignment of lower limbs during a long ride (Abt et al., 2007). Core stability is therefore crucial in injury prevention (Leetun et al., 2004). Targeted core exercises may also contribute to better functional capacity of athletes and enhance their performance.

### 3.2 Gaps in Current Studies Investigating Force-Velocity-Power Characteristics of Exercises Engaging Core Muscles and Proposals for Future Research

Unilateral and/or excessive spine loading for an extended length of time in some sports and physicially demanding professions, or conversely, weakness of the back muscles resulting from a sedentary lifestyle often leads to functional back pain. Recently widely promoted spine stabilization and core strengthening exercises have been seen to reduce these sporting asymmetries on one hand, and improve the strength of back muscles in a sedentary population on the other hand. These exercises have been found to be efficient in the rehabilitation of musculoskeletal injuries, including the lumbar spine, and in the prevention of back pain. However, conflicting and limited evidence exists on their effectiveness for the improvement of athletic performance. This may be ascribed to the lack of a standardized testing battery of core strength and spinal stability. Most current testing methods are not able to identify the likelihood of LBP, they are not sensitive enough in discriminating between and within group differences in these measures, and in revealing their slight changes after exercise programs.

Due to a lack of sport-specific tests, research to date has only marginally addressed the extent to which core strength and spinal stability are associated with athletic performance. The external validity of frequently used isometric and isokinetic tests evaluating maximal muscle strength or muscular endurance of the core for sport-specific activities is ambiguous. Though some studies have demonstrated the relationship between athletic performance and core measures, others have not. Therefore, it is necessary to provide conditions for testing similar to those used during training and competition.

As has been shown, there are significant differences in trunk rotational velocity and power at different weights, as well as force and power at different velocities among athletes of different specializations. Power, velocity, and force produced during rotations of the trunk at a higher or lower velocity, depending on load conditions, are sensitive parameters able to discriminate between athletes with different demands on explosive strength of the core muscles. This testing method can be applied for athletes who require the generation of a high force in a short period of time during trunk rotational movements. This may provide useful data sets for practitioners related to sport-specific group and individual differences in muscle power generated during standing or seated trunk rotations that may be applied for evaluation of the effectiveness of their training programs.

This method may also reveal side-to-side differences in muscle power in asymmetric sports. Taking into consideration small between-side differences in physically active controls (∼7%) and higher power during rotational movements of the trunk on the dominant (D) than non-dominant (ND) side in tennis players (∼12%), ice-hockey players (∼14%) and golfers (∼15%) at different weights, this measure may be considered specific to their asymmetric loading during rotational movements of the trunk in training and competition. However, whether these side-to-side asymmetries, expressed by the D/ND ratio, can predict LBP needs to be investigated.

In practice, maximal strength of the back muscles and their endurance is usually measured not only in athletes but also in the general population, whereas muscle power is often neglected. For instance, poor isometric strength of the back muscles was often associated with LBP. However, this measurement significantly underestimates the loads on the spine during dynamic movements such as lifting tasks. Tests simulating the task being evaluated may be more suitable for individuals with a susceptibility to back problems. Nonetheless, whether this approach would provide deeper insights into understanding the changes in core strength and spinal stability after interventions and across the lifespan of individuals of different ages and level of physical fitness needs to be proven.

Taking into account the vast discrepancy in the methodologies used by different investigations, further research is warranted. It is necessary to quantify kinematic and kinetic variables of exercises involving the use of core muscles, such as lifting tasks or loaded trunk rotations, that are able to reveal between and within-group differences and are sensitive to changes over time. There is a clear need for randomized controlled trials that would verify the efficiency of current testing methods of core strength and spinal stability and their applications in sport and rehabilitation practice.

A recently developed test assessing power during deadlift to high pull simulating the lifting task can be used for this purpose. Preliminary findings revealed that this test is able to reveal training-induced changes in muscle power. However, further studies are needed to investigate the application of this test in functional performance testing of highly skilled athletes and construction workers with high demands on loaded lifting activities or office workers and those with a prevalently sedentary lifestyle. Alternatively, healthy individuals may benefit from this test by predicting the likelihood of LBP.

Further research is also needed to verify the use of RFD measured during the back muscles MVC in subjects who are not able to perform loaded lifting tasks. The strong relationship between the ability to produce a high force in a short period of time and the power generated during a deadlift to high pull exercise suggests that improvements in RFD after training programs may be associated with gains in lifting power at light loads. Given that this measure may be a more suitable predictor of lifting performance than maximal strength of the back muscles, its assessment should be a more appropriate and safer alternative also for LBP prone subjects. Taking into consideration the important role of core stability and core strength in physical fitness and LBP prevention, their evaluation should be a part of the testing battery for both competitive athletes and the general population. Addressing measures that are able to identify back problems resulting from excessive loading of the core or sedentary lifestyle could decrease their future incidence.

So far, a variety of core muscle strength and endurance tests has been designed. However, most of them are not suitable for wheelchair athletes. The core represents for them a basis for production of great power and efficient trunk movements. Limited trunk ROM seems to be responsible for lower velocity of trunk rotations in para table tennis players. Their posterior convexity may also play a role. However, biomechanical factors may also influence the relationship among these measures and have yet to be investigated. Yet further research studies should be conducted to demonstrate the association among lumbar and/or thoracic curvatures, the spine mobility and velocity of trunk rotations. A better understanding of the importance of trunk rotational power and velocity in athletic performance can help us to design exercise programs specific to individual needs.

## 4 Conclusion

This scoping review revealed that among a variety of studies investigating core stability and core strength in sport and rehabilitation, only few of them analyzed the force–velocity–power characteristics of exercises involving the use of core muscles. Most of them evaluated maximal isometric strength of the core and its endurance. However, there were few articles that evaluated muscle power during lifting tasks at different loads performed either with free weights or using the Smith machine. Similarly, power and velocity in the acceleration phase of standing and seated trunk rotations with different weights were evaluated. Nevertheless, there is still scant research investigating the power-velocity and force-velocity relationship during core muscle exercises in able-bodied and para athletes with different demands on stability and strength of the core. Further, there is a lack of papers dealing with the effects of core muscle training on power and velocity produced during resistance exercises engaging core muscles (e.g., deadlift to high pull or wood chop exercise). Therefore, more research is needed to address this gap in the literature and aim research at assessing strength and power-related measures within cross-sectional and intervention studies. A better understanding of the power-force-velocity profiles during exercises with high demands on the core musculature has implications for designing sport training and rehabilitation programs for enhancement of athletes’ performance and/or decrease their risk of back pain.
