# Supplementary material for: Strength and Power-Related Measures in Assessing Core Muscle Performance in Sport and Rehabilitation
Source: Front Physiol. 2022 May 2;13:861582. doi: 10.3389/fphys.2022.861582 (PMC9108269; doi:10.3389/fphys.2022.861582)
Supplement: Supplementary file 1 [file Table1.pdf]

TABLE 1 An overview of studies including basic biomechanical parameters in assessing core muscle performance.

Authors	Study design	Participants	Assessing core muscle strength and power and variables analysed	Main findings
Brown and Abani (1985)	Characteristics of the dead lift of teenage lifters	10 skilled and 11 unskilled contestants in a Michigan Teenage Powerlifting Championship	Equations of motion, force, and moments for a multisegment model of the lifters' movement in the sagittal plane;<br>Body segment orientations, vertical bar accelerations, vertical joint reaction forces, segmental angular accelerations, horizontal moment arms of the bar to selected joints, and intersegmental resultant moments	„Maximum vertical bar acceleration and angular acceleration of the trunk occur near lift-off in the skilled lifters;<br>The unskilled subjects demonstrate greater variability and magnitude in linear and angular acceleration parameters;<br>Maximum vertical force is experienced at the ankle joint;<br>The hip joint experiences the greatest torque because of the relatively large horizontal moment arm of the bar to this joint;<br>The magnitude of the mass lifted, and not the technique, is the primary determinant in the intersegmental resultant moment acting at the hip and the vertical force experienced at the ankle, knee, and hip joints"
Parnianpou et al. (1988)	The effects of isodynamic fatiguing of flexion and extension trunk movements on the movement patterns and the motor output of the trunk	20 male subjects with no history of LBP for the past 6 months	A triaxial dynamometer that simultaneously measure torque, angular position and velocity of each axis;<br>Parameters: trunk motor output and movement patterns; the total angular excursion, range of motion, maximum and average torque and angular velocity of the trunk	„Fatigued muscles are less able to compensate any perturbation in the load or position of the trunk;<br>The repetitive loading results in a weakening of the viscoelastic passive elements of the spineless structure;<br>The loss of ability to protect weakened passive elements makes the spine susceptible to industrial and recreational injuries"
Rosecrance et al. (1991)	A comparison of maximum isometric lifting strength with maximum dynamic lifting capacity	27 men with work-related low back injury	Five dynamic lifting tasks and two isometric strength tests	„There are low to moderate associations between isometric strength measurements and lifting capacity (0.51 with a range of 0.30-0.73) suggesting that estimates of functional lifting capacity should not be based on static measurements alone"

Kumar et al. (1995)	The functional capability in asymmetric trunk motion, isometric and isokinetic lateral flexion and axial rotation strengths	73 normal asymptomatic subjects (41 males and 32 females) and 10 patients (9 males and 1 female)	Isometric lateral flexion tested bilaterally in neutral posture and at 10, 20, and 30 degrees of lateral bending;<br>Isometric axial rotation tested bilaterally in neutral posture and at 5, 10, 15, and 20 degrees of axial rotation;<br>Isokinetic lateral flexion and axial rotation strengths tested bilaterally starting from the neutral posture	„The strength invariably declines progressively with increasing postural asymmetry;<br>The strength in isokinetic activities ranges between 60% to 70% of the strength measured in isometric activities;<br>All groups are significantly stronger in lateral flexion compared with axial rotation“
Kumar (1997)	The isometric and isokinetic axial rotation strengths in neutral and asymmetric postures;<br>The effect of velocity of rotation on the isokinetic trunk strength profile	50 young adults	The isometric strengths measured in neutral, 15 and 30 degrees prerotated trunk postures;<br>The isokinetic strength measured in activities starting from the neutral position to fully rotated and from a fully rotated position to neutral positions at 10, 20, and 40 degrees per second angular velocity	„Participants are significantly stronger in isometric twisting activities than in the isokinetic activities;<br>In isometric activities, participants are 20-25% weaker in prerotated postures when twisting in the direction of prerotation and are approximately 30% stronger in the opposite direction;<br>For isokinetic activities, the trunk rotation from neutral to asymmetric positions produces lesser torques compared with torques from rotated positions to the neutral position;<br>The torque-producing capability declines with increasing velocity of activity“
Stodden et al. (2001)	Variations in pelvis and upper torso kinematics within individual pitchers and their association with variations in pitched ball velocity	19 elite baseball pitchers	3-D high-speed motion analysis;<br>A variation in ball velocity of at least 1.8 m/s (range: 1.8–3.5 m/s) during 10 fastball pitch trials	„Ball velocity within individual pitchers is associated with pelvis orientation at maximum external rotation of the throwing shoulder ($p=0.026$), pelvis orientation at ball release ($p=0.044$), upper torso orientation at maximum external rotation of the throwing shoulder ($p=0.007$), average pelvis velocity during arm cocking ($p=0.024$), and average upper torso velocity during arm acceleration ($p=0.035$);<br>As ball velocity increases, pelvis orientation and upper torso orientation at the instant of maximal external rotation of the throwing shoulder increases;<br>Average pelvis velocity during arm cocking and average upper torso velocity

				during arm acceleration increases as ball velocity increases“
Ellenbecker and Roetert (2004)	Side to side rotational trunk strength	109 elite tennis players	A Cybex isokinetic torso rotation unit at 60 and 120 degrees x s(-1) to measure left and right rotation while stabilized in a seated position	„Elite-level male tennis players have symmetric trunk rotation strength and slightly greater backhand rotation strength (by 4-8%) than forehand rotation“
Iwai et al. (2004)	The relationship between isokinetic trunk muscle strength and the functional disability level of chronic LBP	53 collegiate wrestlers	Trunk extensor and flexor muscle strength measured at three angular velocities (60, 90, and 120 degrees x s(-1));<br>Parameters of trunk muscle strength: peak torque, work, average torque, and average power;<br>The disability level of LBP estimated by using two questionnaires, one developed by Osaka City University (OCU) and the other developed by the Japanese Orthopedic Association (JOA)	„The extensor muscle strength parameters correlate significantly with both the OCU and JOA questionnaires: peak torque at $120°.s^{-1}$ (OCU r=-0.73, JOA r=0.72), work at $60°.s^{-1}$ (OCU r=-0.49, JOA r=0.54) and $90°.s^{-1}$ (OCU r=-0.58, JOA r=0.50), and average torque at $90°.s^{-1}$ (OCU r=-0.63, JOA r=0.47) and $120°.s^{-1}$ (OCU r=-0.73, JOA r=0.73);<br>None of the trunk flexor parameters significantly correlate with the disability level of LBP;<br>The relatively low strength of trunk extensors may be one of the factors related to nonspecific chronic LBP in collegiate wrestlers“
Leetun et al. (2004)	Core stability measures between genders and between athletes who reported an injury during their season versus those who did not;<br>One or a combination of strength measures that could identify athletes at risk for lower extremity injury	80 female intercollegiate basketball and track athletes	Hip abduction and external rotation strength, abdominal muscle function, and back extensor and quadratus lumborum endurance	„Athletes who do not sustain an injury are significantly stronger in hip abduction and external rotation;<br>Hip external rotation strength is the only useful predictor of injury status (OR=0.86, 95% CI=0.77, 0.097);<br>Core stability has an important role in injury prevention“
Lindsay and Horton (2006)	Trunk rotation strength and endurance in healthy individuals who do not play golf and those that are highly skilled at the sport;<br>A comparison of elite golfers with non-debilitating LBP with their healthy counterparts	40 healthy non-golfing control subjects, 32 healthy elite golfers, and 7 golfers with LBP	Bilateral trunk rotation strength and endurance assessed using the Biodex System III Isokinetic Dynamometer with torso rotation attachment;<br>Strength (peak torque) and endurance data	„There are no significant differences in peak torque within or between groups;<br>Golfers with LBP demonstrate significantly less endurance in the non-dominant direction (the follow-through of the golf swing) than either healthy group;<br>There is no significant difference in endurance between the non-golfing controls and the healthy elite golfers;

				There is a poor correlation between the amount of ROM and the amount of torque produced in both dominant (r=-0.29) and non-dominant rotation (r=-0.17);<br>There is a poor correlation between the amount of ROM and the amount of work performed in both dominant (r=0.16) and non-dominant rotation (r=0.36);<br>There is a moderate correlation between body weight and the amount of torque produced in both dominant (r=0.44) and non-dominant rotation (r=0.44);<br>There is a poor correlation between body weight and the amount of work performed in both dominant (r=0.34) and non-dominant rotation (r=0.28)“
Abt et al. (2007)	The relationship between core stability and cycling mechanics of the lower extremity	15 competitive cyclists	Hip, knee, and ankle joint kinematic and pedal force data collected while cycling untethered on a high-speed treadmill;<br>The exhaustive cycling protocol consisting of cycling at 25.8 km x h(-1) while the grade is increasing 1% every 3 minutes;<br>A core fatigue workout performed before the second treadmill test	„Core fatigue results in altered cycling mechanics that might increase the risk of injury because the knee joint is potentially exposed to greater stress;<br>Improved core stability and endurance could promote greater alignment of the lower extremity when riding for extended durations as the core is more resistant to fatigue“
Aguinaldo et al. (2007)	The effects of trunk rotation on shoulder rotational torques during pitching	38 pitchers from the professional, college, high school, and youth ranks	Motion analysis	„Professional pitchers demonstrate the least amount of rotational torque among skeletally mature players, while exhibiting the ability to rotate their trunks significantly later in the pitching cycle, as compared to other groups“
Nesser et al. (2008)	The relationships between core stability and various strength and power variables	29 National Collegiate Athletic Association Division I male strength and power athletes	Strength and performance testing before off-season conditioning;<br>Three strength variables (1RM bench press, 1RM squat, and 1RM power clean), four performance variables (countermovement vertical jump, 20- and 40-yd sprints, and a 10-yd shuttle run), and core stability (back extension, trunk flexion, and left and right bridge)	„Core stability is moderately related to strength and performance;<br>There are significant correlations between total core strength and 20-yd sprint (r=-0.594), 40-yd sprint (r=-0.604), shuttle run (r=-0.551), CMJ (r=0.591), power clean/body weight (BW) (r=0.622), 1RM squat (r=-0.470), bench press/BW (r=0.369), and combined 1RM/BW (r=0.447); trunk flexion and 20-yd sprint

				(r=-0.485), 40-yd sprint (r=-0.479), shuttle run (r=-0.443), CMJ (r=0.436), power clean/BW (r=0.396), and 1RM squat (r=-0.416); back extension and CMJ (r=0.536), and power clean/BW (r=0.449); right bridge and 20-yd sprint (r=-0.410) and 40-yd sprint (r=-0.435), CMJ (r=0.403), power clean/BW (r=0.519) and bench press/BW (r=0.372) and combined 1RM/BW (r=0.406); and left bridge and 20-yd sprint (r=-0.376) and 40-yd sprint (r=-0.397), shuttle run (r=-0.374), and power clean/BW (r=0.460)"
Balague et al. (2010)	The association between isoinertial trunk muscle performance and consequential (non-trivial) LBP	95 healthy male adolescents	Anthropometric measurements, clinical evaluation, and tests of trunk ROM, maximum isometric strength and peak movement velocity using an isoinertial device	„Regular involvement in sport is a consistent predictor of LBP;<br>Isoinertial trunk performance is not associated with LBP in adolescents"
Keogh et al. (2010)	Determining whether a range of static core stability measures could distinguish shoulder press performance in unstable vs. stable conditions	30 resistance-trained men	1RM strength (from <6 repetitions) predicted in the seated shoulder dumbbell press performed in unstable (Swiss ball) and stable (back-support bench) environments;<br>Three CS muscle endurance tests with 4 CS ratios calculated;<br>The degree of strength decrement, referred to as the instability strength level, calculated by dividing the predicted 1RM Unstable score by the 1RM Stable score	„There are no significant between-group differences (p=0.132–0.999) or large effect sizes for any of the core stability measures;<br>Trunk flexion endurance is the only core stability measure that significantly correlates to the instability strength level (r=0.477);<br>Core stability exhibits relatively high levels of task specificity, thus core stability performance in static single-joint exercises may not be highly related to that in more dynamic multijoint activities"
Palmer and Uhl (2011)	The interday reliability of peak muscular power outputs while participants performed diagonal chop and lift tests and maintained a stable trunk	18 healthy individuals (10 men and 8 women) from the general population	Two power tests (chop, lift) using an isotonic dynamometer and three endurance tests (Biering-Sørensen, side-plank left, side-plank right);<br>Main outcome measures: peak muscular power outputs derived from a 1RM protocol for the chop and lift tests collected for both the right and left sides	„ICCs for peak muscular power are highly reliable for the chop (0.87-0.98), lift (0.83-0.96), and endurance (0.80-0.98) tests between test sessions;<br>The correlations between the power assessments and the Biering-Sørensen test are low (-0.008 to 0.017);<br>The side-plank tests are moderately correlated with the chop (0.528-0.590) and the lift (0.359-0.467) tests"

Sharrock et al. (2011)	The relationship between core stability and athletic performance measures	35 student athletes from Asbury College (NAIA Division II)	A series of five tests: double leg lowering (core stability test), the forty yard dash, the T-test, vertical jump, and a medicine ball throw	„Medicine ball throw negatively correlates to the core stability test (r=–0.389); Participants that perform better on the core stability test have a stronger negative correlation to the medicine ball throw (r=–0.527)“
Andre et al. (2012)	The test-retest reliability of kinetic and kinematic rotational characteristics of a pulley system when performing a rotational exercise of the axial skeleton in the transverse plane	Healthy, college-aged men (n=8) and women (n=15)	Torso rotations while sitting on a box, 3 times per trial, with 3 loads: 9% body weight (BW), 12% BW, and 15% BW;<br>The repetition with the greatest power for each trial for each load was analyzed	„When the participants are separated by sex, there are no significant differences between groups;<br>The ICC for mean peak power repetition is 0.97 (9%BW), 0.94 (12%BW), and 0.95 (15%BW);<br>A pulley system and an external dynamometer can be used together as a reliable research tool to assess rotational power“
Aytar et al. (2012)	The relationship between core stability, balance and strength	11 amputee soccer players	A Kinesthetic Ability Trainer device used to assess balance;<br>Trunk strength evaluated by isokinetic dynamometer;<br>Gillet test used to evaluate the sacroiliac joint movement;<br>Modified plank test used to determine dynamic core stability	„There is a significant correlation between flexor isokinetic trunk muscle strength at the velocity of 60°/sec and modified plank test (r=0.630);<br>There is a significant negative correlation between flexor isokinetic trunk muscle strength at the velocity of 180°/sec and Oswestry Disability Index score (r=-0.649)“
Shinkle et al. (2012)	Developing a functional field test assessing the role of the core musculature and its impact on sport performance in an athletic population and a functional field test determining how well the core can transfer forces from the lower to the upper extremities	25 DI collegiate football players	Medicine ball throws (forward, reverse, right, and left) in static and dynamic positions;<br>Athletic performance measurements: 1RM squat, squat kg/bw, 1RM bench press, bench kg/bw, countermovement vertical jump (CMJ), 40-yd dash (40 yd), and proagility (PrA);<br>Push press power (PWR) for measuring the transfer of forces through the body	„Static reverse correlates with CMJ (r=0.44), 40 yd (r=0.50), and PrA (r=0.46);<br>Static left correlates with bench kg/bw (r=0.42), CMJ (r=0.44), 40 yd (r=0.62), and PrA (r=0.59);<br>Static right correlates with bench kg/bw (r=0.41), 40 yd (r=0.44), and PrA (r=0.65);<br>Dynamic forward correlates with the 1RM squat (r=0.45) and 1RM bench (r=0.41);<br>Dynamic left and Dynamic right correlate with CMJ (r=0.48 and r=0.40, respectively);<br>Push press power correlates with bench kg/bw (r=0.50), CMJ (r=0.48), and PrA (r=0.48);

				A stepwise regression for PWR prediction identifies 1RM squat as the best predictor“
Fan et al. (2014)	Trunk muscle strength and EMG activation during isokinetic axial rotation at different angular velocities	24 healthy young men	Isokinetic axial rotation in right and left directions at 30, 60, and 120 degrees per second angular velocity;<br>Surface EMG recorded on external oblique, internal oblique, and latissimus dorsi bilaterally	„Velocity of rotation has great impact on the axial rotation torque and EMG activity;<br>There is an inverse relationship of angular velocity with the axial rotation torque and root mean square value of individual trunk muscle;<br>Higher velocity is associated with higher coactivation of antagonist, leading to a decrease in torque with the increase of velocity“
Talukdar et al. (2015)	The role of rotational power and mobility on cricket ball-throwing velocity	11 professional cricketers and 10 under-19 club-level cricketers, divided into 2 groups (fast and slow) based on their standing cricket ball-throwing velocity	The chop and lift, seated and standing cricket ball throw, seated and standing side medicine ball throw, and seated active thoracic rotation ROM and hip rotation ROM	„The seated and standing cricket ball throws on the dominant side are significantly different between fast and slow throwers;<br>Muscular performance measures, such as bilateral thoracic rotation ROM, hip external rotation ROM on the dominant side, and force and work required in the chop, are significantly different between fast and slow throwers;<br>Faster throwers display greater force and work outputs in the chop compared with the slower throwers; however, slower throwers show significantly greater ROM in the thoracic and hip regions“
Barbado et al. (2016))	The influence of specialization in sports with large but qualitatively different balance control demands on trunk stability;<br>A comparison high-performance athletes with recreational athletes without a specific training history	25 judokas, 16 kayakers and 37 recreational athletes	Two trunk stability protocols: sudden loading to assess trunk responses to external and unexpected perturbations; stable and unstable sitting to assess the participant's ability to control trunk while sitting	„Specific-sport training induces specific trunk stability adaptations, which are not revealed through nonspecific tests“
Freeston et al. (2016)	The relationship between measures of strength and/or power and throwing velocity	17 male cricket players from an elite athlete program	Maximal throwing velocity from the stretch position and after a 3-meter shuffle;<br>Strength and power using a range of different measures	„There is a link between strength and/or power and throwing velocity in cricket players;<br>Throwing velocity from the stretch position is significantly related to

				dominant leg lateral-to-medial jump distance (r=0.71), dominant shoulder internal rotation strength (r=0.55), and dominant (r=0.73) and nondominant (r=0.54) medicine ball rotation throw velocity and medicine ball chest pass distance (r=0.67); There is a significant correlation between velocity after a shuffle step and vertical jump height (r=0.51); Exercises that more closely simulate the speed (body weight jumps and medicine ball throws) or movement pattern (shoulder internal rotation) of overhead throwing are greater predictors of throwing velocity“
Zemková et al. (2016)	The reliability of data obtained from deadlift to high pull on the Smith machine and with free weights; A comparison of peak and mean values of power during deadlift to high pull with different weights using the Smith machine and free weights; The association between the power produced during the deadlift to high pull and the deadlift and the upright row, respectively	32 healthy young, predominantly sedentary men who performed very little physically activity, undergoing only obligatory exercise courses at the university	Deadlift to high pull with increasing weights, and deadlift and upright row with a previously established weight of 70% 6RM, either using a Smith machine or free weights; Peak and mean values of power were analysed	„Peak and mean power are significantly higher during deadlift to high pull with free weights than on the Smith machine, however only at ≥50 kg; Their maximal values are achieved at about 80% and 70% 1RM respectively; The deadlift to high pull with free weights is a reliable test with ICC >0.80 and SEM <10% for mean power; It is also a sensitive test distinguishing lifting performance among healthy young individuals“
Zemková et al. (2017a)	The repeatability and sensitivity of a novel method for assessing the maximal power during the standing cable wood chop exercise with different weights and the endurance of the core muscles	23 fit men with experience with resistance training, including exercises to strengthen the trunk muscles	Maximal effort single repetitions of the standing cable wood chop exercise with weights increasing stepwise up to 1RM; A set of 20 repetitions at a previously established weight at which maximal power was achieved; Mean values of power were analyzed	„Mean power during the standing cable wood chop exercise is a reliable parameter, with ICC above 0.90 for all weights tested; It is a sensitive parameter able to discriminate within-group differences in the maximal power and endurance of core muscles, especially at higher weights“
Zemková et al. (2017b)	Peak and mean power during standing and seated trunk	27 fit men, all of them with ~3.5 years’ experience	Four trials of trunk rotations in both standing and seated positions with a bar weight of 5.5,	„Both peak power and mean power in the acceleration phase of trunk rotations are significantly higher during standing than

	rotations with different weights	with resistance training including exercises strengthening the trunk muscles, and no active in any sport at a competitive level that would require loaded trunk rotations	10.5, 15.5, and 20 kg placed on the shoulders;<br>Peak and mean values of velocity in the acceleration phase of trunk rotation as well as respective angular displacements were analysed	seated trunk rotations at weights of 20 kg, 15.5 kg, and 10.5 kg but not at 5.5 kg;<br>Peak and mean power during standing trunk rotations significantly correlates with values achieved in the seated position at the weight of 5.5 kg (r=0.684 and r=0.676 respectively) but not at 10.5 kg (r=0.589 and r=0.552 respectively), 15.5 kg (r=0.493 and r=0.436 respectively), and 20 kg (r=0.357 and r=0.333 respectively)“
Kocahan and Akınoğlu (2018)	The relationship between core endurance and isokinetic muscle strength of knees and shoulders	71 elite athletes (weight lifting, boxing, taekwondo, biathlon, and ice skating)	Isokinetic muscle strength of shoulder internal-external rotation and knee flexion-extension determined by using an Isomed 2000 device;<br>The Mcgill Core Endurance Tests	„There is a relationship between the shoulder internal rotation and external rotation peak torque/body weight (PT/W) and all endurance tests except extension endurance tests;<br>There is a relationship between knee flexion PT/W and all core endurance tests;<br>There is a relationship between knee extension PT/W and extension endurance and the lateral bridge test, but not with the flexor endurance test“
Zemková et al. (2018a)	The relationship between peak and mean velocity during trunk rotations and respective angular displacement	91 young and older subjects of both genders	Five rotations of the trunk to each side in a seated position with a barbell of 1 kg and 20 kg placed on their shoulders behind the neck;<br>Peak and mean values of angular velocity from the acceleration phase of trunk rotation and angular displacement were analysed	„Peak and mean velocity in the acceleration phase of trunk rotations, as well as trunk angular displacement are significantly higher in young than older adults with both 1 kg and 20 kg;<br>Peak and mean velocity correlates significantly with a range of trunk rotational motion at both weights in young (0.650-0.790) and older adults (0.772-0.927)“
Zemková et al. (2018b)	The relationship of trunk rotational velocity with mobility and curvature of the spine in wheelchair athletes	11 male para table tennis players and 13 gender and age-matched able-bodied athletes;<br>Wheelchair athletes: 7 quadriplegics	Five repetitions of trunk rotations to each side in the seated position with a barbell of 1 kg placed on their shoulders behind the neck;<br>Peak and mean values of angular velocity and acceleration from the acceleration phase of trunk rotation, as well as respective angular displacement were analysed;	„Peak and mean velocity of trunk rotation and respective angular displacement are significantly lower in para table tennis players compared to able-bodied athletes;<br>Both groups show similar values of thoracic kyphosis;<br>Para table tennis players exhibit lower lumbar inversion and pelvic retroversion compared to able-bodied athletes;

		(TT2) / tetraplegics (TT3) and 4 paraplegics (TT4); Para table tennis players classified into these classes: 2 (n=5), 3 (n=3), and 4 (n=3) based on the classification system of the International Table Tennis Federation; Able-bodied athletes from sports, in which trunk rotations play an essential role in performance (over 10 years' experience in a particular sport with at least 6 years' experience in a competition)	Spine curvature was measured using a Spinal Mouse system (Idiag, Fehraltdorf, Switzerland)	Para table tennis players exhibit lower thoracic and lumbar mobility during maximal trunk flexion in the seated position with flexed knees compared to able-bodied athletes; Para table tennis players have a greater pelvic tilt than able-bodied athletes; Range of trunk rotational motion significantly correlates with the peak and mean velocity in the acceleration phase of trunk rotation in both para table tennis players (r=0.912 and r=0.819 respectively) and able-bodied athletes (r=0.790 and r=0.673 respectively); Trunk rotational velocity values are associated with lumbar curvature (r=−0.787 and r=−0.713 respectively) and pelvic tilt angle (r=0.694 and r=0.746 respectively) in para table tennis players but not in able-bodied athletes"
Michaelides et al. (2019)	Abdominal strength in professional soccer players and comparison to their lower body strength	132 professional male soccer players from Cyprus's first and second divisions	A lower body isokinetic test and an isometric abdominal test; Three and twenty-five maximal concentric flexion and extension repetitions at angle speeds of 60°/s and 300°/s respectively	„Abdominal strength has low to moderate significant correlations with quadriceps and hamstrings strength at both isokinetic speeds of 300°/s and 60°/s; The variability in isokinetic variables accounted for only 14-16% of the variability of abdominal strength; Abdominal strength appears to be high in professional soccer players, but is not dependent on the sports level and/or a playing position"
Shaikh et al. (2019)	The relationship of the core power and endurance with variables of athletic performance (T test, medicine ball throw test, vertical jump test and 40 yard dash test) in random intermittent dynamic type sports	58 male collegiate athletes involved in intermittent dynamic type sports	Core power, core endurance and performance tests; The core endurance measured by McGill protocol and double leg lowering test and the core power measured by 60 seconds maximal sit-up test	„McGill protocol is positively correlated with medicine ball throw test (r=0.688) and vertical jump test (r=0.463); There is a strong negative correlation of McGill protocol with 40 yard dash test (r=-0.525) and T-test (r=-0.687);

				Double leg lowering test is positively correlated with 40 yard dash test (r=0.374) and T-test (r=0.524);<br>Only medicine ball throw test is related significantly with the tests of core power;<br>Core muscle endurance is necessary for optimal performance“
Zemková (2019)	The test-retest reliability of trunk rotational power and velocity over a 1-week interval using the Torso Isoinertial Dynamometer	32 physically active men with experience with resistance training including exercises strengthening the trunk muscles	Five trunk rotations to each side while seated with a barbell of 1 kg or 20 kg placed on their shoulders;<br>Peak and mean power, peak and mean angular velocity, peak and mean force, and mean angular displacement were analysed	„Peak and mean velocity in the acceleration phase of trunk rotations with 1 kg provides reliable results (ICC=0.94 and 0.92 respectively, SEM=7.0% and 7.3% respectively);<br>Peak and mean values of velocity and power obtained during trunk rotations with a weight of 20 kg should be interpreted with caution (ICC <0.80, SEM >10%)“
Zemková et al. (2019a)	Peak and mean values of power during trunk rotations on the dominant and non-dominant side	17 golfers, 17 ice-hockey players, 21 tennis players, and 39 age-matched control group of physically active individuals	Standing trunk rotations to each side with a bar weight of 5.5, 10.5, 15.5, and 20 kg placed on the shoulders;<br>Peak power and mean power in the acceleration phase of trunk rotations were analysed	„The power is significantly higher during trunk rotations on the dominant than non-dominant side in golfers (~15%), tennis players (~12%) and ice-hockey players (~14%) at lower and/or higher weights, whereas there are no significant side-to-side differences in a control group of fit individuals (~7%)“
García-Vaquero et al. (2020)	The reliability and the learning effect of an isokinetic trunk flexion-extension protocol designed to simultaneously assess trunk muscle strength and endurance	57 healthy and physically active young men (n=28) and women (n=9)	Four trials of 15 maximum flexion-extension concentric exertions at 120°/s (range of trunk motion = 50°);<br>The absolute and relative peak torque and total work to assess trunk flexion and extension strength;<br>Endurance ratio, modified endurance ratio, fatigue final ratio, recovery ratio, and modified recovery ratio variables to assess trunk muscle endurance in both directions	„A 10-min single-session protocol provides a reliable muscle strength and endurance evaluation of trunk flexor and extensor muscles, all within the same protocol“
Zemková et al. (2020)	Power and velocity at different loads, and power and force at different velocities during trunk rotations in athletes of different sports	23 grappling sports athletes (judo, wrestling), 39 combat sports athletes (boxing, thai boxing, karate, tae kwon do), 52 ball	Standing trunk rotations on each side with bars of different weights (from 1 kg up to 50 kg) placed on the shoulders;<br>Power, angular velocity and force were analysed	„There are significant between-group differences in mean power in the acceleration phase of trunk rotations, especially at higher weights (≥10.5 kg) or lower velocities (≤334.2 rad/s);<br>The power at 10.5 kg is significantly higher in combat than water, grappling and

		sports athletes (golf, hockey, tennis) and 19 water sports athletes (canoeing, kayaking)		ball sports athletes, with no significant differences between the two latter groups; at 15.5 kg, it is higher in water than grappling and ball sports athletes but not those in combat sports; and at 20 kg, it is higher in water than grappling and ball sports athletes, with no significant differences with those in combat sports“
de Bruin et al. (2021)	The relationship between core stability and athletic performance	83 female athletes (hockey, netball, running, soccer and tennis)	The isometric back extension (IBE), lateral flexion (LF) and abdominal flexion (AF) tests to measure core strength and endurance;<br>The core stability grading system using a pressure biofeedback unit to measure core neuromuscular control (NMC);<br>Athletic performance assessed using the 40 m sprint, T-test, vertical jump (VJ) and the medicine ball chest throw (MBCT)	„Most correlations are weak (r=0.10–0.39), although there is a very strong correlation between LF (strength) and VJ (r=0.90);<br>There are moderate correlations (r=0.40–0.69) between core strength, endurance and motor control with certain athletic performance tests in all five sport codes;<br>In runners, strong correlations (r=0.70–0.89) are between AF (endurance) and VJ, and in tennis players between IBE (strength) and the sprint“
